# The DIAMIND study: postpartum SMS reminders to women who have had gestational diabetes mellitus to test for type 2 diabetes: a randomised controlled trial – study protocol

**DOI:** 10.1186/1471-2393-13-92

**Published:** 2013-04-12

**Authors:** Emer Heatley, Philippa Middleton, William Hague, Caroline Crowther

**Affiliations:** 1 Australian Research Centre for Health of Women and Babies, Robinson Institute, The University of Adelaide, 72 King William Road North, Adelaide, SA 5006, Australia; 2 The Liggins Institute The University of Auckland, Private Bag 92019, Victoria Street West, Auckland 1142, New Zealand

**Keywords:** Gestational diabetes mellitus, Reminder system, SMS text reminder, Randomised controlled trial, Postpartum care, Oral glucose tolerance test, Type 2 diabetes mellitus

## Abstract

**Background:**

Postpartum follow up of women who have been found to have gestational diabetes during pregnancy is essential because of the strong association of gestational diabetes with subsequent type 2 diabetes. Postal reminders have been shown to increase significantly attendance for oral glucose tolerance testing postpartum. It is possible that a short message service (text) reminder system may also be effective. This trial aims to assess whether a text message reminder system for women who have experienced gestational diabetes in their index pregnancy will increase attendance for oral glucose tolerance testing within six months after birth.

**Methods/Design:**

*Design*: Single centre (Women’s and Children’s Hospital, South Australia), parallel group randomised controlled trial.

*Inclusion criteria*: Women diagnosed with gestational diabetes in their index pregnancy (oral glucose tolerance test with fasting glucose ≥ 5.5 mmol/L and/or two hour glucose ≥ 7.8 mmol/L), with access to a mobile phone, whose capillary blood glucose profile measurements prior to postnatal discharge are all normal (fasting glucose < 6.0 mmol/L, postprandial glucoses < 8.0 mmol/L).

*Exclusion criteria*: Pregestational diabetes mellitus, triplet/higher order multiple birth or stillbirth in the index pregnancy, requirement for interpreter.

*Trial entry and randomisation*: Allocation to intervention will be undertaken using a telephone randomisation service (computer-generated random number sequence generation, with balanced variable blocks, and stratification by insulin requirement).

*Study groups*: Women in the intervention group will receive a text reminder to attend for an oral glucose tolerance test at 6 weeks postpartum, with further reminders at 3 months and 6 months if they do not respond to indicate test completion. Women in the control group will receive a single text message reminder at 6 months postpartum.

*Blinding*: Baseline data collection will be undertaken blinded. Blinding of participants and blinded collection of primary outcome data will not be possible for this study.

*Primary study outcome*: Attendance for the oral glucose tolerance test within 6 months postpartum.

*Sample size*: 276 subjects will be required to show an 18% absolute increase in the rate of attendance (α=0.05 two tailed, β=80%, 5% loss to follow up) from 37% to 55% in the intervention group.

**Discussion:**

Given the heightened risk of impaired glucose tolerance and type 2 diabetes in women who have had gestational diabetes, ensuring the highest possible rate of attendance for postpartum glucose tolerance testing, so that early diagnosis and intervention can occur, is important. A text message reminder system may prove to be an effective method for achieving improved attendance for such testing. This randomised controlled trial will assess whether such a system will increase rates of attendance for postpartum oral glucose tolerance testing in women who have experienced gestational diabetes.

**Trial Registration:**

Australian New Zealand Clinical Trials Registry - ACTRN12612000621819

## Background

### Introduction

Gestational diabetes mellitus (GDM) has been defined as carbohydrate intolerance of variable severity with onset or first recognition during pregnancy [1]. GDM affects 5% of pregnancies in Australia, and the prevalence of GDM is likely to rise with increases in maternal age and obesity [2]. Women who have had GDM are at higher risk for development of type 2 diabetes mellitus (DM) in the future compared with women who have had normal blood glucose values during pregnancy, as well as being at increased risk of GDM in future pregnancies [3-7]. Therefore, many clinical practice guidelines recommend screening for type 2 DM and impaired glucose tolerance in the postpartum period [8-13]. Such follow up is important, given the well-documented risks to women and their babies resulting from type 2 DM and GDM [14], the availability of interventions for prevention of type 2 DM and recurrent GDM such as lifestyle changes, use of metformin, and encouragement of breastfeeding [15], and because direct medical costs increase greatly with progression from impaired glucose tolerance to type 2 DM with complications [16]. However, follow up rates for GDM tend to be sub-optimal and need to be improved [17]. Increasing the rate of follow up with an oral glucose tolerance test (OGTT) is important, given that it is the most sensitive test for detection of impaired glucose tolerance and type 2 DM within 6 months postpartum [18].

### Reminder systems for increasing postpartum follow up of GDM

Reminder systems have been used to improve healthcare with positive results, and some research studies have assessed the effect of reminders on increasing attendance for follow up of GDM. For example, implementation of a postpartum patient reminder system for women who had experienced GDM into routine care at two hospital sites in Canada (The Ottawa Hospital, General Campus and the Queensway Carleton Hospital) resulted in higher rates of completion of oral glucose tolerance testing by 6 months postpartum, with a rate of 28% (41/145) compared with 14% (16/117 women) in the site where reminders were not used (The Ottawa Hospital, Civic Campus) (p = 0.01) [19].

However, the rate of completion in both sites where reminders were used was considerably lower than the rates of OGTT completion observed in the randomised controlled trial (RCT) of postal reminders previously conducted by the same team at The Ottawa Hospital in Canada [20]. In the RCT, OGTT completion rates were 60% (49/81 women) in the physician and patient reminder group, 55% (42/76 women) in the patient-only reminder group, 52% (16/31) in the physician-only reminder group and 14% (5/35 women) in the group with no reminders sent (p < 0.05). It should be noted that OGTT completion was measured up until six months postpartum in the implementation study, compared with up to one year postpartum in the RCT.

A small number of non-randomised studies have examined reminder systems for postpartum blood glucose testing in women who have experienced GDM. A Finnish prospective observational study found that a phone call reminder by a nurse increased rates of postpartum oral glucose tolerance testing at one year after birth (odds ratio 13.4, 95% confidence interval 4.6–38.1) [21]. Another study examined the efficacy of a checklist as a physician reminder for increasing postpartum screening for type 2 DM in women who had GDM at the Endocrine Obstetrics Clinic of the Women’s College Hospital in Toronto, Canada. In this study, retrospective chart review revealed that use of the reminder checklist for physicians was associated with a 3 fold increase in odds of a woman being screened with an OGTT, as measured at ≥ 6 months postpartum (odds ratio 2.99, 95% confidence interval 1.84–4.85) [22].

Following on from a previous study investigating trends in postpartum glucose test ordering by clinicians and completion by women (with recent GDM) that found increasing but suboptimal rates at Kaiser Permanente Northwest (KPNW, a large non-profit health organisation in western Oregon and Washington state) [23], another study at KPNW examined the efficacy of several interventions aimed at increasing the proportion of postpartum glucose test ordering by clinicians and test completion by women (including revising the nursing protocol for pregnant women with GDM, improving the electronic medical record system, educating clinical staff and providing additional reminders to women who did not complete the test within 3 months of delivery) [24]. Orders for postpartum glucose screening increased from 77% of (155/200) women in the pre-implementation period, to 89% (159/179) in the post-implementation period (p = 0.004), and completion of postpartum glucose screening increased from 60% (pre-implementation) to 72% (post-implementation) (hazard ratio, 1.37; 95% confidence interval, 1.07–1.75).

### Postpartum reminders for GDM follow up in Australia

The South Australian Gestational Diabetes Recall Register was established in July 2002 [25]. This register sent a reminder letter at 15 months after birth to women who had experienced GDM to encourage oral glucose tolerance testing. A study examining the efficacy of the register found that, of the 429 women who had been sent their first reminder letter (at 15 months), 56% had undertaken a glucose test for diabetes (response rate 46%) [25]. There was considerable variation in the rate of recruitment of eligible women to the register, with a nadir of 27% in 2006, and a peak of 72% in 2003. The authors of this study speculated that reasons for this variability in recruitment rates may have included time constraints during appointments, change in staff, and differences in staff efforts to recruit to the register. The South Australian register has now been replaced by a national register, the National Gestational Diabetes Register [26]. Women resident in Australia and eligible for a Medicare card are recruited to this register at the time of registering for the National Diabetes Services Scheme (NDSS) following a diagnosis of GDM, and the register sends a follow-up reminder letter to such women to visit a general practitioner to arrange an OGTT at 12–16 weeks after the expected due date (provided to the register at the time of registration) and an information booklet called *Life after Gestational Diabetes*.

### Short message service (SMS) reminders – the reminders of the future?

In Australia in 2011, there were at least 28 million mobile phone subscriptions (i.e. 6 million more subscriptions than people) [27]. With the very high rate of mobile phone usage, it is likely that almost all women of reproductive age have access to mobile phones. Given the high rate of mobile phone use in Australia, and the low cost of SMS messages, a reminder system that utilises SMS technology might prove to be a cost-effective way of increasing the number of women in Australia with a recent history of GDM, who could then be prompted to undertake oral glucose tolerance testing in the postpartum period. Several studies in other areas of health care have indicated that SMS reminders can increase appointment attendance rates [28-31]. This potential to increase rates of attendance may translate into increased rates of oral glucose tolerance testing in the postpartum period for women who have had GDM.

### Aims and objectives

The primary aim of this RCT is to determine whether an SMS reminder system will significantly increase attendance for oral glucose tolerance testing by 6 months postpartum in women who have recently experienced GDM.

### Hypotheses

The primary hypothesis is that a SMS reminder system for women who have recently had GDM will increase the number of women who complete oral glucose tolerance testing by 6 months postpartum.

## Methods/Design

### Ethics statement

Ethics approval was obtained from the Women’s and Children’s Health Network Human Research Ethics Committee (REC2200/8/2015).

### Study design

Single centre (Women’s and Children’s Hospital, South Australia), parallel group randomised controlled trial.

### Inclusion criteria

Women diagnosed with GDM in their index pregnancy (positive 75 g OGTT with fasting glucose ≥ 5.5 mmol/L and/or two hour glucose ≥ 7.8 mmol/L), with access to a personal mobile phone, whose capillary blood glucose profile measurements prior to hospital discharge after giving birth are normal (fasting blood glucose < 6.0 mmol/L and 2 hour postprandial blood glucoses < 8.0 mmol/L), who provide written, informed consent, will be included in the trial.

### Exclusion criteria

Pregestational diabetes mellitus, triplet/higher order multiple birth or stillbirth in the index pregnancy or requirement for interpreter.

### Trial entry

Women who are potentially eligible for the study will be approached in the postnatal ward, counselled and given the study information sheet. They will be entered into the trial if they give written consent and have normal blood glucose profile results prior to discharge from hospital.

### Study groups and management

Eligible women will be randomised into one of two study groups: either the ‘6 week (intervention) group’ or the ‘6 month (control) group’.

### Randomisation

Randomisation will be carried out using a telephone randomisation service. The randomisation schedule has balanced variable blocks and has been prepared by an investigator not involved in recruitment or clinical care. Randomisation will be stratified by antenatal requirement for drug therapy to treat GDM.

### Treatment schedules

#### Intervention (6 week reminder) group

Women in the intervention group will be sent a SMS reminder at six weeks after the birth of their baby: “*Hi* (*Participant Name*), *This is a reminder from the DIAMIND study team for you to have your oral glucose tolerance test for type 2 diabetes*. *Please let us know when you have done the test*, *and what the results were by texting us on* (*study number*) *or emailing us at* (*study email address*) - *Thanks for participating and best wishes*”. If the participant responds to say she has completed the test, no further text reminders will be sent. If not, a further text reminder will be sent at three and six months (same message).

### Control (6 month reminder) group

Women in the control group will receive no text reminders for the first 6 months of the study period. A single text message reminder (same text as for intervention group) will be sent to these women at 6 months postpartum (measured from date of birth of baby).

### Primary study outcomes

Oral glucose tolerance test undertaken by 6 months postpartum

### Secondary study outcomes

Fasting blood glucose test undertaken by 6 months postpartum

Glycated haemoglobin (HbA1c) test undertaken by 6 months postpartum

### Data collection

#### Baseline data collection

Baseline data will be collected to assess the similarity between the two groups in terms of factors that may influence attendance for postpartum oral glucose tolerance testing. At trial entry, information will be collected on demographic characteristics of the participants as well as smoking history, current BMI at booking and previous pregnancy outcomes. With regards to GDM, women will be asked whether or not they were given the opportunity to join the National Diabetes Services Scheme (NDSS) and therefore the National Gestational Diabetes Register, whether they joined, and where they intend to have their postpartum OGTT completed. Data regarding control of GDM (dietary control only or requirement for metformin or insulin), diagnostic OGTT date and results, and complications at birth relating to GDM (requirement for induction, caesarean section, perineal injury, blood loss) will also be collected.

Baseline data relating to the health of the newborn(s) will be collected: singleton or twin, birth order (if twin), gestational age at birth, birth weight (grams), time of birth, gender, Apgar scores, mode of birth (normal vaginal birth, operative vaginal birth, caesarean section), nerve palsy, bone fracture, newborn hypoglycaemia (plasma glucose ≤ 2.0 mmol/L), neonatal intensive care admission, respiratory distress syndrome, neonatal jaundice requiring phototherapy, and death prior to first discharge.

The following information will be collected at hospital discharge: breastfeeding status (given the link between breastfeeding and reduced risk of type 2 DM [32,33], as well as the possible influence that breastfeeding may have on the mother’s ability to attend for oral glucose tolerance testing [34]), mention of GDM in the problem list of the discharge summary and whether or not follow up oral glucose tolerance testing was recommended in the discharge summary.

### Outcome data collection: 6 months postpartum

All women in the study will be asked to complete a questionnaire at 6 months after the birth of the baby either by post or by email (using Survey Monkey), depending on their expressed preference at trial entry, to ascertain whether an OGTT was undertaken within the first 6 months, or whether a fasting blood glucose or glycated haemoglobin (HbA1c) test was used instead. The questionnaire also asks for the date and results of these tests, where known. The date and results of the OGTT will be confirmed using the hospital clinical information system, or by contact with the participant’s general practitioner, where necessary. The questionnaire also examines women’s attitudes towards the OGTT, as well as reasons for not being able to undertake the test, if applicable. Where questionnaires are not returned within 2 weeks, participants will be contacted by telephone, and then again in another 2 weeks if the questionnaires are still not received by the study team. During this telephone contact, participants will be asked whether or not they have completed an OGTT within the six months since they gave birth.

### Sample size

The baseline rate of OGTT uptake used in the sample size calculation for the proposed trial is 37%; this rate is at the lower end of the range in the recent review by Tovar and colleagues (2011) [17]. Using a figure from the lower end of this range is a sound estimate, given that the health centres in the study had reminder systems in place, and that some results came from surveys with moderately low response rates [17]. Data from the South Australian Gestational Diabetes Mellitus Recall Register indicates that the actual rate may be somewhere between 26 and 56 percent [25].

The Stata version 10.0 sample size calculator has been used to calculate the target sample size.

The figures entered were:

▪ baseline uptake of postnatal OGTT 37%

▪ projected 18% absolute improvement to 55% (48% relative increase)

▪ power 80%

▪ significance (two-tailed) 5%

This resulted in a calculation that the study will require 262 women (131 in each arm). With a predicted up to 5% loss to follow up, 276 women will be required.

### Analyses and reporting of results

Baseline characteristics of all randomised women will be compared descriptively between the study groups. Outcome comparisons will be made according to the treatment allocation at randomisation on an ‘intention to treat’ basis. Categorical variables will be reported as risk ratios with corresponding 95% confidence intervals. Continuous outcomes will be reported as mean (and standard deviation) for normally distributed results, or median (interquartile range) for results which are not normally distributed. All model assumptions will be assessed. Statistical significance will be defined at the 0.05 level using a two-sided comparative test.

## Discussion

This randomised controlled trial of a SMS reminder system to improve the rate of attendance for follow up oral glucose tolerance testing in women who have experienced GDM is important given the high, and rising, rate of GDM, and the strong association between GDM and subsequent type 2 DM worldwide. In Australia, this is a particularly timely trial, given the recent establishment of the National Gestational Diabetes Register, the increasingly ubiquitous use of mobile phones, and the decreasing use of postal services (“snail mail”) for communication. Such a reminder system may prove to be a cost-effective measure to reduce future rates of type 2 DM in Australian women, and is therefore of public health as well as obstetric importance. Such a system has the potential to be implemented locally or on a wider scale to improve the health of all women who have experienced GDM.

## Abbreviations

DM: Diabetes mellitus; GDM: Gestational diabetes mellitus; HbA1c: Haemoglobin A1c; OGTT: Oral glucose tolerance test; RCT: Randomised controlled trial; SMS: Short message service.

## Competing interests

The author(s) declare that they have no competing interests.

## Authors’ contributions

EH, PM, WH and CAC are all members of the DIAMIND Study Group. The primary investigator of the DIAMIND Study (EH) prepared the initial draft of the DIAMIND protocol. All members of the DIAMIND study team participated in the design of the study. The DIAMIND Study Group participated in the protocol development, commented on drafts of the protocol, and have read and approved the final draft of the protocol. All authors read and approved the final manuscript.

## Pre-publication history

The pre-publication history for this paper can be accessed here:

http://www.biomedcentral.com/1471-2393/13/92/prepub
